# A Complex Case of Pulmonary Silico-Tuberculosis and Review of Literature

**DOI:** 10.3390/diagnostics13172728

**Published:** 2023-08-22

**Authors:** Lauren D’Sa, Federica Pezzuto, Francesca Lunardi, Francesca Scalvenzi, Mariaenrica Tinè, Giovanni Comacchio, Chiara Giraudo, Fiorella Calabrese

**Affiliations:** 1 Department of Histopathology, Addenbrooke’s Hospital, Cambridge CB20QQ, UK; lauren.dsa@nhs.net; 2 Department of Cardiac, Thoracic and Vascular Sciences, University of Padova, 35128 Padova, Italy; federica.pezzuto@unipd.it (F.P.); francesca.lunardi@unipd.it (F.L.); francesca.scalvenzi@studenti.unipd.it (F.S.); mariaenrica.tine@unipd.it (M.T.); gcomacchio@gmail.com (G.C.); 3 Department of Medicine, University of Padova, 35128 Padova, Italy; chiara.giraudo@unipd.it

**Keywords:** pulmonary silico-tuberculosis, histology, microbiology, molecular

## Abstract

Silicosis caused by the inhalation/deposition of free silica particles is characterized by pulmonary inflammation/fibrosis. Among the clinical disorders associated with silicosis, tuberculosis is by far the most prominent. A 66-year-old male non-smoker, originally from North Africa, reported a dry cough and significant weight loss. He was a foundry worker. He had a medical history of bladder carcinoma associated with schistosomiasis. Computed tomography (CT) and positron emission tomography (PET)/CT showed bilateral multiple hypermetabolic lung nodules, some with cavitation. The patient underwent surgical resection of the largest nodule, which was highly suspicious of lung metastasis. The histological examination revealed multiple nodular formations. Several lesions showed the characteristic features of silicotic nodules. There were also adjacent well-formed granulomas, some with central caseous necrosis. A real-time polymerase chain reaction, performed for the identification and quantification of the DNA of the *Mycobacterium tuberculosis* complex, was positive. Pulmonary silico-tuberculosis is often encountered in patients with a history of silica exposure in tuberculosis-endemic areas. This case serves as a reminder to never underestimate patient occupational exposure and geographic origin. A careful histological diagnosis and molecular investigation are mandatory when approaching difficult cases, especially patients with a prior cancer history and clinical/radiological features suggestive of tumour recurrence/metastasis.

The deposition of free crystalline silica particles in the lungs can cause irreversible pulmonary fibrosis. Silicosis is largely an occupational-related disease seen in industries such as mining, foundries, sandblasting, and glassworks. Despite a World Health Organisation strategy to eliminate silicosis by 2030 [1], in 2017, 23,695 incident silicosis cases were reported, mainly concentrated in developing countries [2].

The exact pathogenesis of silicosis is incompletely described. There is evidence of alveolar macrophage impairment following the uptake of silica crystals. This is mediated by reactive oxygen species and lysosomal damage with a subsequent downstream proinflammatory and pro-fibrotic effect [3]. Silicosis is also associated with an increased risk of additional localised and systemic conditions, such as lung cancer, autoimmune conditions, and infections [3]. Autoimmune conditions include systemic sclerosis, systemic lupus erythematosus, and rheumatoid arthritis. Caplan syndrome describes the radiographic appearance of multiple well-defined pulmonary nodules that develop in patients with rheumatoid arthritis and pneumoconiosis, such as silicosis [4].

Silicosis can be complicated by mycosis [5], however, more widely known is the association of silicosis and mycobacterial infections. A study on gold miners showed that there was an increased risk of both pulmonary and extrapulmonary mycobacterial tuberculosis in men with silicosis [6]. The pleura is the most common extrapulmonary site [7]. Nontuberculous infections have also been identified [8]. Silico-tuberculosis can be a challenging diagnosis due to overlapping radiological appearances, such as sarcoidosis, lung cancer, and hypersensitivity pneumonitis [3]. This report aims to highlight that pneumoconioses can present unexpectedly, especially in patients with a more complex medical history.

We report a case of a 66-year-old male non-smoker, originally from North Africa and living in Italy for more than 25 years. He worked for a short period as a farmer and then as a foundry worker. He had a history of urinary schistosomiasis and bladder squamous cell carcinoma for which he underwent a radical cystectomy. He was undergoing radiological and urinary surveillance for the recurrence of bladder carcinoma.

Small lung lesions appeared on computed tomography (CT) scanning approximately 5 years after his radical cystectomy. CT and positron emission tomography (PET) imaging over subsequent years showed the lesions evolved into bilateral hypermetabolic lung nodules, some with cavitation, as shown in Figure 1.

During this period, the patient reported weight loss and a dry cough but denied dyspnoea. Scheduled follow-up visits were delayed during the COVID-19 pandemic and when it was possible, the patient underwent a fine needle biopsy with high suspicion of tumour metastasis. Blood investigations showed haemoglobin (Hb) 133 g/L, a white cell count (WCC) 4.64 × 10^9^/L, and platelets 164 × 10^9^. Coagulation, renal function, and liver function investigations were within normal limits. Tumour markers were negative. Quantiferon testing was positive. The largest lung mass was resected, and the histology is described in Figure 2. Microdissection of the largest necrotizing granuloma was performed for the molecular investigation of mycobacteria. A real-time polymerase chain reaction, performed for the identification and quantification of the DNA of the *Mycobacterium tuberculosis* complex, was positive. On later genotyping, the rpoB gene mutations conferring rifampicin resistance were absent. The patient was finally diagnosed with a silico-tuberculosis.

A new bronchoscopy was performed for microbiologic analysis. The Ziehl–Neelsen stain was positive, and *Mycobacterium tuberculosis* grew on bronchial aspirates, so the patient started with antituberculosis polytherapy (isoniazid, rifampin, pyrazinamide, and ethambutol). A pulmonary function test documented a moderate obstructive ventilatory deficit (FEV 1 66% of predicted, FEV 1/FVC 0.6), and an anticholinergic inhaler, umeclidinium bromide, was prescribed with a beneficial effect on the mild dyspnoea.

Among the clinical disorders associated with silicosis, *Mycobacterium tuberculosis* (MTB) is by far the most prominent. Alveolar macrophage impairment following silica exposure promotes an increased susceptibility for mycobacterial growth [9]. Studies have shown silica particles facilitate the intracellular replication and release of MTB from macrophages [10]. The role of the adaptive immune response is less clear.

Silicotic nodules are usually up to 5 mm in dimension, although they can coalesce to form larger areas of fibrosis. They predominate in the upper lobes. The architectural lung changes due to chronic silicosis are irreversible. Anti-tuberculosis chemotherapy can be given as a preventative or treatment for latent or active MTB in patients with silicosis, however, there are no recent guidelines that standardise the chemotherapy in silico-tuberculosis. Rupani (2023) [11] retrospectively compared the outcomes of patients in Gujarat, India with silico-tuberculosis against those with tuberculosis but without silicosis, following anti-tuberculous chemotherapy. The silico-tuberculosis patients had 2.3 times higher odds of unfavourable treatment outcomes and a higher likelihood of relapse. A higher risk of relapse was also identified within a population of South African gold miners who received isoniazid, pyrazinamide, and streptomycin and rifampicin for five days a week for five months [12]. Rupani recommended that all patients with silicosis and a positive screen for tuberculosis be treated with an anti-tuberculous regime for 6 months (for drug-sensitive MTB) ranging to 20 months (for drug-resistant MTB). An extended course of chemotherapy of at least 8 months for patients with silicosis has also been recommended [13].

To further explore the literature on the diagnosis of silico-tuberculosis, a search of case reports on silico-tuberculosis was performed on 22 May 2023. The PubMed search included case reports written in English using the search terms ‘lung silicotuberculosis’ or ‘lung silico-tuberculosis’ or ‘lung silicosis’ and ‘lung tuberculosis’. The search returned 64 results, of which 44 were excluded for reasons including an inaccessible report, non-tuberculosis infections, silicosis without tuberculosis, and formats other than case reports [14,15,16,17,18,19,20,21,22,23,24,25,26,27,28,29,30,31,32,33]. The 20 included case reports described 26 relevant individual cases whose major findings are reported in Table 1.

We have defined ‘silico-tuberculosis’ as including both active and latent MTB infections. Where possible, the investigations from the first presentation/diagnosis of silico-tuberculosis are provided and case reports detailing follow-up visits are indicated.

The listed case reports highlight that the issue of silico-tuberculosis is longstanding and is not limited to developing countries. A thorough medical history is required during the assessment of patients who have emigrated, for both the consideration of MTB (as in our case) but also the recognition of higher-risk occupations.

In our case, we preferred to investigate mycobacteria by using molecular investigation. We did not perform acid-fast stains for the assessment of mycobacteria as acid-fast staining has shown a lower sensitivity in formalin-fixed paraffin-embedded tissue compared to other methods [34]. The molecular analysis of MTB has shown a high sensitivity [35] and can be rapidly performed, compared to the 6–8-week time frame of culture methods. A further advantage of molecular analysis is the ability to quickly test for resistance to anti-tuberculosis therapeutics that can influence the pharmacological approach. In our case, the rpoB gene mutation conferring rifampicin resistance was negative. Rifampicin mono-drug resistance is unusual, and instead usually occurs alongside isoniazid resistance. The assessment of rpoB can therefore act as a surrogate marker of multidrug resistance [36]. For these reasons, we suggest that, where resources allow, PCR should always be performed on FFPE tissue. Many of the case reports rely only on radiology and investigations confirming latent/active MTB without histopathological analysis. Chronic silicosis presents with bilateral, nodular opacities. Cavitation is highly suspicious for MTB infection. Unlike the other cases listed, our case is unique in having a more complex medical history. Primary lung/metastatic tumours can also show cavitation. This made relying heavily on radiological findings difficult and the pathology was essential in reaching the correct diagnosis. Earlier consideration of histopathology is, therefore, important, as is a multidisciplinary approach integrating microbiological, radiological, and pathological investigations.

## Figures and Tables

**Figure 1:**
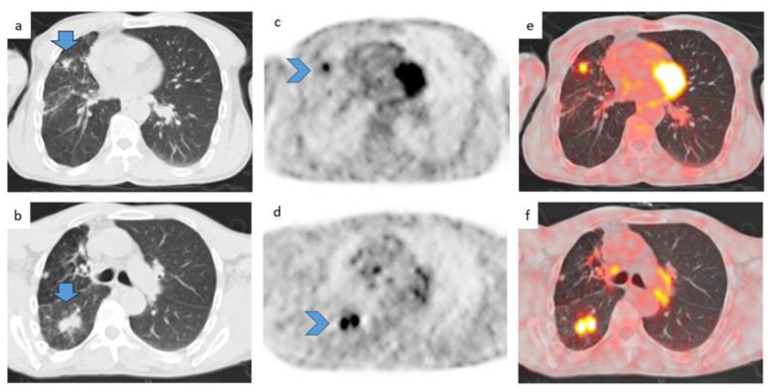
Axial CT images with a pulmonary window of the whole body showing multiple pulmonary nodules (arrows) (**a**,**b**) with significant tracer uptake (arrowheads) (**c**,**d**). Fused axial images in (**e**,**f**).

**Figure 2:**
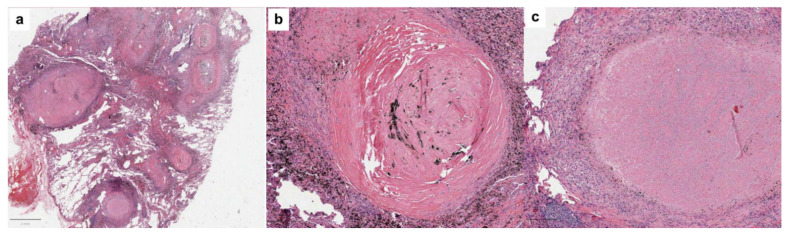
Histology showed well-circumscribed small nodules, mainly localized in subpleural and peribronchial sites ((**a**), scale bar: 2 mm). Several nodules showed characteristic features of silicotic nodules with a central zone of cellular hyalinized collagen with a whorled appearance and a peripheral zone of dust-laden macrophages ((**b**), scale bar: 200 µm). Polarized light microscopy showed bright white crystals of varying sizes. There were adjacent well-formed granulomas, some with central caseous necrosis ((**c**), scale bar: 200 µm). There was no evidence of malignancy. All special stains for microorganisms (bacteria and fungi: PAS, Gram, and Grocott) were negative.

**Table 1:** Summary of the case reports on silico-tuberculosis described in the literature.

Author	Country	Age, Sex	Medical History	Histology Performed	Results of Diagnostic Tests for TB			
					Tuberculin Skin Test	Acid-Fast Staining	Culture	PCR
Maboso et al. (2023) [14] ^†^	South Africa	61, M		None				
Maboso et al. (2023) [14] ^†^	South Africa	67, M		None				
Maboso et al. (2023) [14]	South Africa	73, M		Biopsy				-
Khemakhem et al. (2022) [15]	Tunisia	23, M		Lung biopsy		-		
Djatioetomo et al. (2022) [16]	Indonesia	28, M		None				+
Skowroński et al. (2018) [17] ^†^	Poland	58, M		None				-
Lefkowitz et al. (2017) [18]	Syria	44, M		TBBx		-		+
Anderson et al. (2016) [19]	Australia	72, M	IHD and hypothyroidism	None		+		
Nicolini et al. (2016) [20] ^†^	Italy	71, M		None				+
Rai et al. (2016) [21]	India	43, M		None		+		
Murlidhar et al. (2015) [22]	India	11, M	Malnourishment	None		+		
Oni et al. (2015) [23]	South Africa	59, M		None				
Sureka et al. (2013) [24]	India	46, M		Lung biopsy	+	+		
Tung et al. (2013) [25]	Hong Kong	74, M		EBUS-TBNA			+	
Ozden et al. (2012) [26]	Turkey	21, M		None	-	-		+
Milovanović et al. (2011) [27]	Serbia	52, M		None				
Martins et al. (2010) [28] ^†^	Brazil	55, M		None		-	+	
Scafa et al. (2004) [29]	Italy	72, M		None	+	-		
Scafa et al. (2004) [29] ^†^	Italy	84, F		None			+	
Ho et al. (2003) [30] ^†^	Hong Kong	71, M		Lung biopsy		+		
Safa et al. (2003) [31]	Saudi Arabia	50, F	Arthritis and diabetes	TBBx		-	-	
Morgan (1979) [32]	USA	58, M		None	+			
Morgan (1979) [32]	USA	39, M		None				
Kawakami et al. (1977) [33]	Japan	53, F		None	+	+		
Kawakami et al. (1977) [33]	Japan	45, M		None	+	-	-	
Kawakami et al. (1977) [33]	Japan	58, M	Pleurisy 50 years ago	None	+	-	-	

Abbreviations: M: male, F: female, IHD: ischemic heart disease, SOB: shortness of breath, SOBOE: shortness of breath on exertion, EBUS-TBNA: endobronchial ultrasound-transbronchial needle aspiration, TBBx: transbronchial biopsy, ^†^ follow up visit with known silicosis or tuberculosis and not the primary presentation.

## Data Availability

Not applicable.
